# Changes in vital signs during adrenaline administration for hemostasis in intracordal injection: an observational study with a hypothetical design of endotracheal adrenaline administration in cardiopulmonary arrest

**DOI:** 10.1186/s13019-023-02376-1

**Published:** 2023-10-06

**Authors:** Tomohiro Hasegawa, Yusuke Watanabe

**Affiliations:** Tokyo Voice Center, International University of Health and Welfare, 8-5-35 Akasaka, Minato-ku, Tokyo, 107-0052 Japan

**Keywords:** Advanced cardiovascular life support, Basic fibroblast growth factor, Cardiopulmonary arrest, Endotracheal adrenaline administration, Intracordal injection under local anesthesia

## Abstract

**Background:**

The background is that intravenous adrenaline administration is recommended for advanced cardiovascular life support in adults and endotracheal administration is given low priority. The reason is that the optimal dose of adrenaline in endotracheal administration is unknown, and it is ethically difficult to design studies of endotracheal adrenaline administration with non-cardiopulmonary arrest. We otolaryngologists think so because we administered adrenaline to the vocal folds for hemostasis after intracordal injection under local anesthesia, but have had few cases of vital changes. We hypothesized that examining vital signs before and after adrenaline administration for hemostasis would help determine the optimal dose of endotracheal adrenaline.

**Methods:**

We retrospectively examined the medical records of 79 patients who visited our hospital from January 2018 to December 2020 and received adrenaline in the vocal folds and trachea for hemostasis by intracordal injection under local anesthesia to investigate changes in heart rate and systolic blood pressure before and after the injection.

**Results:**

The mean heart rates before and after injection were 83.96 ± 18.51 (standard deviation) beats per minute (bpm) and 81.50 ± 15.38 (standard deviation) bpm, respectively. The mean systolic blood pressure before and after the injection were 138.13 ± 25.33 (standard deviation) mmHg and 135.72 ± 22.19 (standard deviation) mmHg, respectively. Heart rate and systolic blood pressure had *P*-values of 0.136, and 0.450, respectively, indicating no significant differences.

**Conclusions:**

Although this study was an observational, changes in vital signs were investigated assuming endotracheal adrenaline administration. The current recommended dose of adrenaline in endotracheal administration with cardiopulmonary arrest may not be effective. In some cases of cardiopulmonary arrest, intravenous and intraosseous routes of adrenaline administration may be difficult and the opportunity for resuscitation may be missed. Therefore, it is desirable to have many options for adrenaline administration. Therefore, if the optimal dose and efficacy of endotracheal adrenaline administration can be clarified, early adrenaline administration will be possible, which will improve return of spontaneous circulation (ROSC) and survival discharge rates.

## Background

Adrenaline, which is indicated in cases of cardiopulmonary arrest, can be administered endotracheally. However, the evidence presented by the Japan Resuscitation Council Resuscitation Guidelines 2020 [1] includes the comparison of the efficacy of intravenous and intraosseous routes of adrenaline administration in secondary resuscitation of adults, with no mention of endotracheal administration. In addition, the American Heart Association (AHA) [2] and European Resuscitation Council (ERC) [3] recommend, in order of priority, the administration of resuscitation drugs through intraosseous and endotracheal routes when intravenous administration is not possible, indicating the low priority of endotracheal administration. The evidence is based on animal and cardiopulmonary arrest patient experiments by Niemann et al. [4], Quinton et al. [5], and Burgert et al. [6], which are difficult to verify in human with non-cardiopulmonary arrest for medical ethical reasons.

In the field of otorhinolaryngology head and neck surgery, adrenaline may be administered to the vocal folds for hemostasis after intracordal injection under local anesthesia. In our hospital, after intracordal injection, adrenaline (Bosmin^®^ 0.1% solution; Daiichi Sankyo Co. Ltd., Tokyo, Japan) is administered directly to the vocal folds, the dose being 0.67 mg of adrenaline. By contrast, the adrenaline kit (Adrenaline Injection^®^ 0.1% syringe; Terumo Co. Tokyo, Japan) 1 ampule, which is administered intravenously to patients with cardiopulmonary arrest, contains 1 mg of adrenaline in 1 mL which is at most 1.5 times higher than the dose of adrenaline used for hemostasis after our intracordal injection. In our experience, however, endotracheal adrenaline administration for hemostasis after the injection rarely results in a sudden immediate increase in heart rate or systolic blood pressure.

From the above, we concluded that the reasons for the low priority of endotracheal adrenaline administration in cardiopulmonary arrest are: (1) it is ethically difficult to design a prospective clinical study with a high level of evidence for endotracheal adrenaline administration in humans under non-cardiopulmonary arrest, and (2) in our experience, we have never experienced a sudden change in vital signs when adrenaline is administered around the vocal folds, so the dosage of adrenaline is small, i.e., the optimal endotracheal adrenaline dose is unknown.

Therefore, we hypothesized that the administration of adrenaline for hemostasis in intracordal injection is similar to the endotracheal adrenaline administration in non-cardiopulmonary arrest. We investigated medical records to retrospectively analyze the changes in heart rate and systolic blood pressure before and after the administration of adrenaline for hemostasis in intracordal injection under local anesthesia in our institution. Thus, we investigated the extent to which endotracheal adrenaline administration to vocal folds affected the heart rate and systolic blood pressure in human with non-cardiopulmonary arrest, and to test the appropriateness of the endotracheal adrenaline dose in cardiopulmonary arrest.

## Methods

### Study design

Retrospectively study using medical records. This study was carried out in accordance with ‘WMA Declaration of Helsinki 2013–Ethical Principles for Medical Research Involving Human Subjects’ and our national research guideline ‘Ethical Guidelines for Medical and Health Research Involving Human Subjects’.

### Study participants

**Fig. 1:**
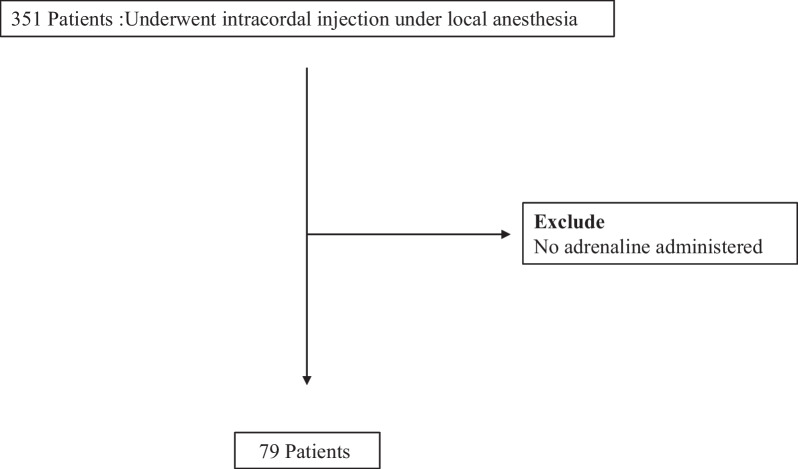
Study enrollment. A total of 351 patients underwent intracordal injection under local anesthesia. Patients who did not require adrenaline to stop bleeding were excluded, and 79 patients were studied

All 351 patients had blood samples, electrocardiograms, and chest radiographs taken prior to injection to ensure that they were in a general condition to tolerate the injection procedure. And they were performed intracordal injection after confirming that they had no history of hepatic disorders, cardiovascular or respiratory diseases, other serious systemic diseases, other laryngeal diseases, laryngeal cancer, or drug allergies. Of the 351 patients, 79 had bleeding from the vocal cords after injection and adrenaline was administered to the vocal cords for hemostatic purposes (Fig. 1). The 79 patients consisted of 20 men and 59 women, with a mean age of 48.6 (range, 18–82) years (Table 1).

**Table 1:** Patient background characteristics

	Number	%
Sex		
Male	20	25
Female	59	75
Age		
-29	14	18
30–39	13	16
40–49	8	10
50–59	25	32
60–69	12	15
70-	7	
Average	46.8	
Diagnosis		
Chorditis	27	34
Nodules	16	20
Polypoid	11	14
Scar	8	10
Atrophy	6	8
Bamboo node	3	4
Edema	3	4
Paralysis	2	2
Others	3	4
Drug		
Tr-A	72	91
Trafermin	7	9

*Tr-A* triamcinolone acetonide

### Injection procedure

**Fig. 2:**
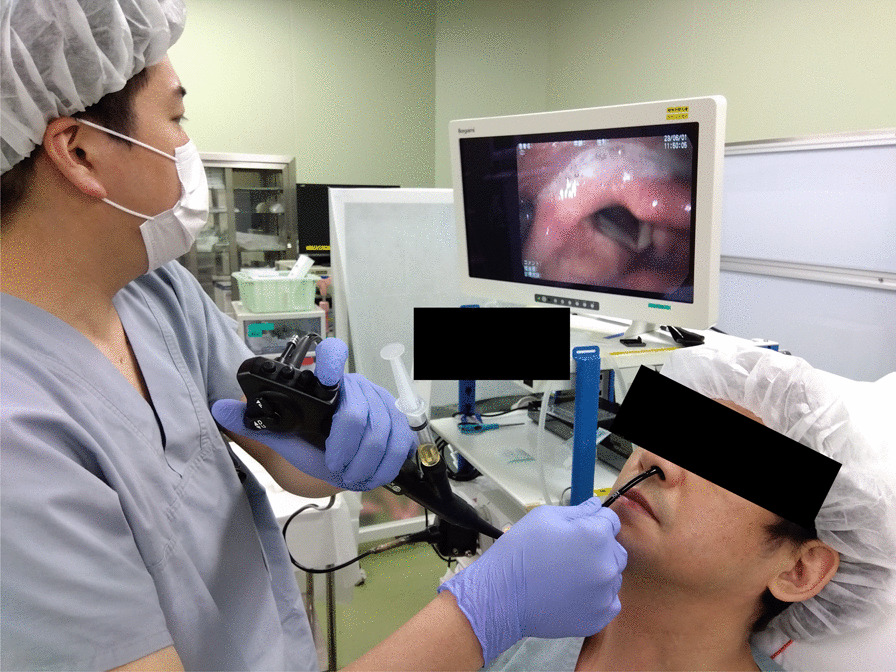
Intracordal injection under local anesthesia method. A laryngeal endoscope is inserted through the nostrils to observe the vocal folds

**Fig. 3:**
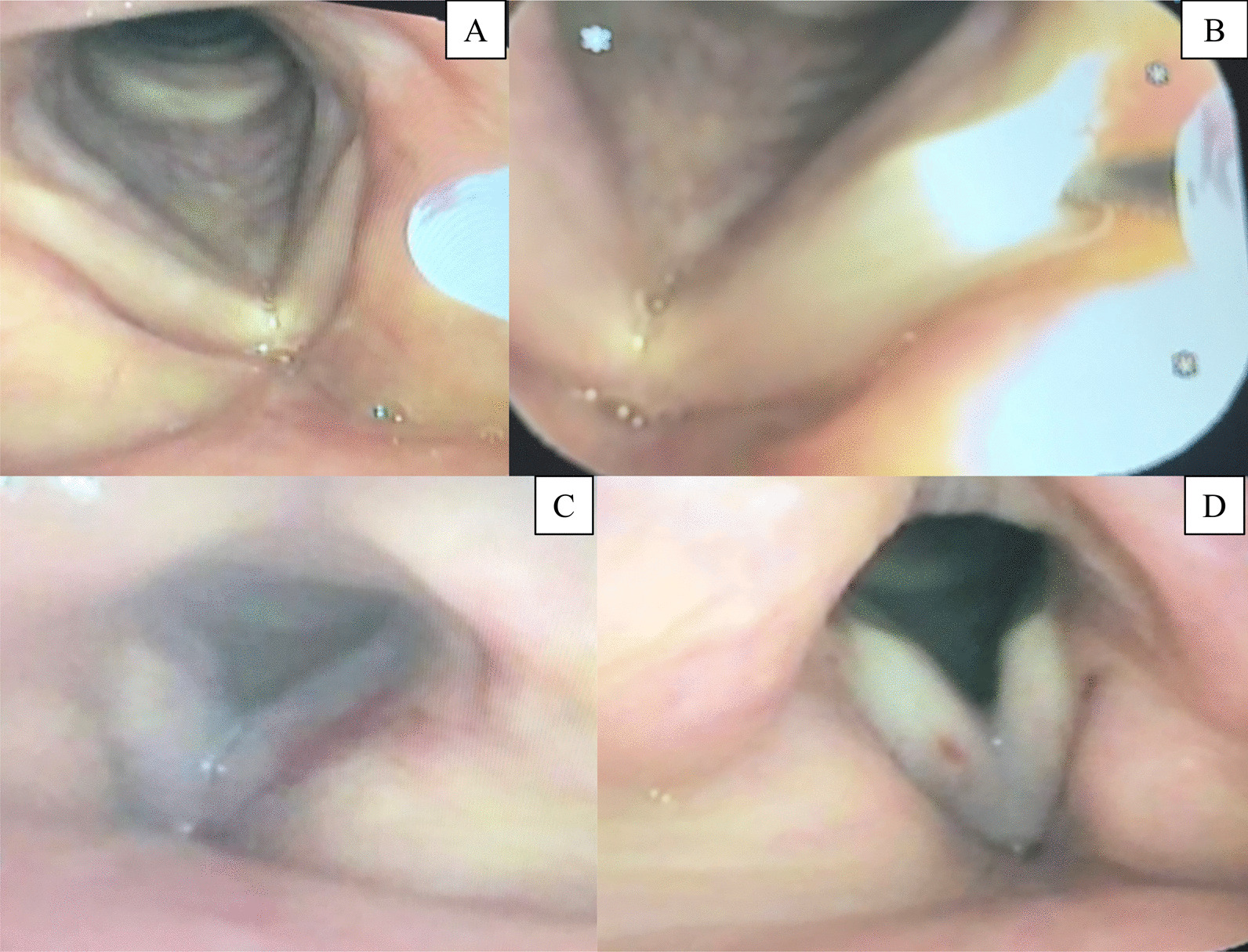
Intracordal injection under local anesthesia and hemostasis with adrenaline. **A** Vocal folds after local anesthesia by dropping 4% lidocaine hydrochloride hydrate onto the larynx. **B** The injection into the vocal folds using a 23-gauge esophageal varicose vein-indwelling needle. **C** and **D** After injection, when bleeding is observed in the vocal folds, a dose of 3000-fold diluted adrenaline is dropped directly onto the vocal folds for hemostasis

The intracordal injection under local anesthesia is shown in Figs. 2 and 3. The local anesthesia method first involved the insertion of a 4% lidocaine hydrochloride hydrate (4% Xylocaine^®^, Aspen Japan Co. Ltd., Tokyo, Japan)-soaked gauze into both nostrils for 30 min to anesthetize the nose. Afterward, the patient was moved to the operating room, where laryngeal anesthesia was performed by dropping 4% lidocaine hydrochloride hydrate into the larynx using a laryngeal endoscope while in a sitting position. The sites of laryngeal anesthesia included the epiglottis at the base of the tongue, epiglottis at the laryngeal side, vocal folds, and arytenoid portion. A 23-gauge esophageal variceal indwelling needle (Varixar^®^; Top Co., Ltd., Tokyo, Japan) was used to inject two drugs into the right and left vocal folds: triamcinolone acetonide (Kenacort-A^®^; Bristol Myers Squibb, Tokyo, Japan), and trafermin (Fibrast^®^; Kaken Pharmaceutical Co., Tokyo, Japan). After injection, when bleeding was observed in the vocal folds, a dose of 3000-fold diluted adrenaline (0.1% solution, Bosmin^®^, Daiichi Sankyo Co. Ltd., Tokyo, Japan) was dropped directly onto the vocal folds with vocalization for hemostasis. The composition of the adrenaline was 100 mL of adrenaline diluted with 200 mL of saline solution to make a total volume of 300 mL, of which 2 mL was used only once for hemostasis.

### bFGF preparation

Trafermin is basic fibroblast growth factor (bFGF) preparation. Trafermin is used as a dermatological spray to treat bedsores and skin ulcers and in emergency medicine to treat burns. In the field of voice medicine, trafermin injection into the vocal folds has been reported to improve voice disorders in diseases such as vocal fold atrophy, scarring, paralysis, and sulcus. Several institutes have reported the usefulness of intracordal trafermin injection in improving voice [7–9]. In this study, we dissolved 100 μg of trafermin per 1 mL of the attached dissolution solution and injected 50 μg into each vocal folds. The intracordal trafermin injection is considered off-label use. We received approval from the Ethics Committee (with approval number). The off-label use was subsequently approved, and the injections were conducted as a self-pay after explaining the procedure to all patients and obtaining their written informed consent.

### Heart rate and systolic blood pressure measurement method

**Fig. 4:**
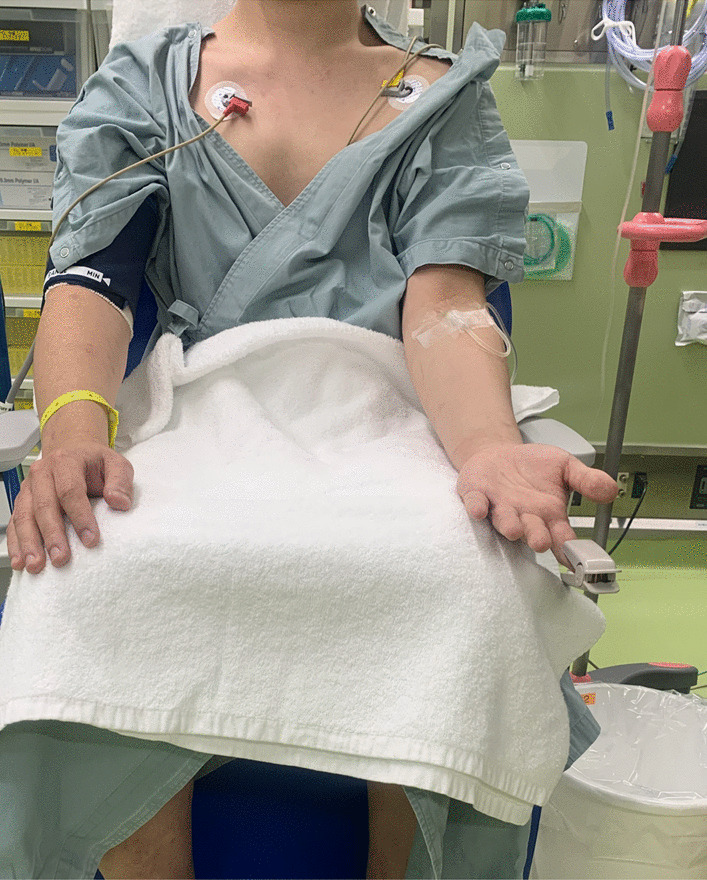
Heart rate and systolic blood pressure measurement method. An automatic sphygmomanometer is attached to the upper arm where the peripheral venous route is not secured. A continuous electrocardiogram monitor is attached to the chest

The methods of measuring heart rate and systolic blood pressure are shown in Fig. 4. For blood pressure measurements, upon entering the operating room, an automatic sphygmomanometer was attached to the upper arm of the patient on the side where the peripheral venous route was not secured, and the systolic blood pressure values were measured before and after intracordal injection. For heart rate measurements, a continuous electrocardiogram monitor was used to record the heart rate during blood pressure measurement before and after injection.

### Statistical analyses

The Wilcoxon signed rank test was used because changes in inspection results before and after surgery were for the same subject in the comparative test, thus the data did not follow a normal distribution. *P* < 0.05 was set as statistically significant.

## Results

The Table 2 shows the changes in heart rate and systolic blood pressure. In heart rate, 7 patients had a decrease of 20 or more after injection compared to pre-injection, 10 patients had a decrease between 10 and 19, 23 patients had a decrease between 1 and 9, 29 patients had an increase between 0 and 9, 8 patients had an increase between 10 and 19, and 2 patients had an increase of 20 or more.

**Table 2:** The changes in heart rate and systolic blood pressure before and after the intracordal injection

Heart rate (bpm)	Number	Systolic blood pressure (mmHg)	Number
~ − 20	7	~ − 20	7
− 19 ~ − 10	10	− 19 ~ − 10	12
− 9 ~ − 1	23	− 9 ~ − 1	22
0 ~ 9	29	0~9	21
10 ~ 19	8	10~19	14
20 ~	2	20 ~	3

In systolic blood pressure, 7 patients had a decrease of 20 or more after injection compared to pre-injection, 12 patients had a decrease between 10 and 19, 22 patients had a decrease between 1 and 9, 21 patients had an increase between 0 and 9, 14 patients had an increase between 10 and 19, and 3 patients had an increase of 20 or more.

**Fig. 5:**
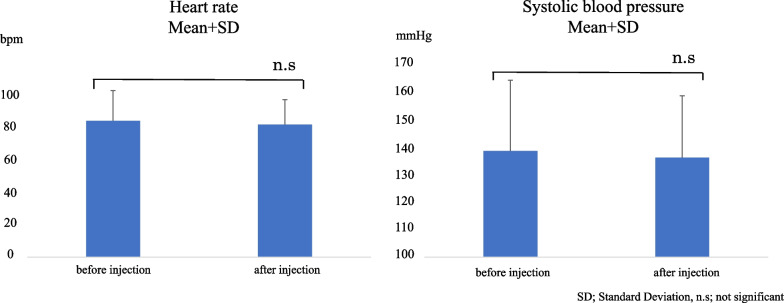
Statistical analysis results. The mean heart rates before and after the injection are 83.96 ± 18.51 and 81.50 ± 15.38 bpm, respectively. The mean systolic blood pressures before and after the injection are 138.13 ± 25.33 and 135.72 ± 22.19 mmHg, respectively. The P-values for heart rate and systolic blood pressure are 0.136 and 0.450, respectively, and they show no significant differences

The mean changes in heart rate and systolic blood pressure before and after the injection in the 79 adrenaline-treated patients are shown in Fig. 5. The mean duration of the injection was 3.01 min. The mean heart rates before and after the injection were 83.96 ± 18.51 (standard deviation) beats per minute (bpm) and 81.50 ± 15.38 (standard deviation) bpm, respectively. The mean systolic blood pressure before and after the injection were 138.13 ± 25.33 (standard deviation) mmHg and 135.72 ± 22.19 (standard deviation) mmHg, respectively. The *P*-values for the differences in heart rate and systolic blood pressure were 0.136 and 0.450, respectively, indicating no significant differences.

## Discussion

In this study, we used medical records to retrospectively investigate the changes in heart rate and systolic blood pressure in 79 patients who underwent intracordal injection under local anesthesia and received adrenaline for hemostasis at our hospital. The results showed no significant difference between before and after adrenaline administration.

### Trends in indications for endotracheal adrenaline administration in cardiopulmonary arrest

Currently, the intravenous administration of adrenaline is the most recommended method of administration in cases of cardiopulmonary arrest. The efficacy of endotracheal adrenaline administration in cardiopulmonary arrest is inferior to that of intravenous. In the endotracheal adrenaline administration, Redding et al. [10] in 1967 evaluated the efficacy of endotracheal adrenaline in cardiopulmonary arrest by comparing various routes of adrenaline administration for resuscitation of dogs with cardiac arrest due to airway obstruction. The results showed that the endotracheal administration of diluted adrenaline was not inferior in efficacy to intravenous or intracardiac administration, and the endotracheal administration of adrenaline was recommended. In 1979, Roberts et al. [11] also demonstrated absorption of adrenaline from the lungs. In recent years, however, in adults, the AHA and ERC have recommended that resuscitation drugs be administered by the intraosseous route or endotracheally, in the same order of preference, when intravenous administration is not possible. Niemann et al. [4] reported that endotracheal adrenaline was associated with lower hospital discharge rates and return of spontaneous circulation (ROSC) in comparison with intravenous administration in adult cardiopulmonary arrest. Quinton et al [5] reported that endotracheal adrenaline was unreliable for out-of-hospital cardiopulmonary arrest. In 2019, Burgert et al. [6] reported the pharmacokinetic effects of endotracheal, intraosseous, and intravenous adrenaline in a porcine traumatic cardiac arrest model and argued that the absorption rate of endotracheal adrenaline administration was variable and less reliable than that of intravenous and intraosseous administration. Thus, at present, endotracheal adrenaline administration is not a priority in the treatment of cardiopulmonary arrest, based on the results of such animal studies or observational studies in cardiopulmonary arrest. On the other hand, it is difficult to design a study in which endotracheal adrenaline is administered in human with non-cardiopulmonary arrest.

### Adrenaline to vocal folds for hemostasis

In the field of otorhinolaryngology head and neck surgery, endoscopic examinations and injections are commonly performed. In the field of voice medicine, drugs are injected directly into the vocal folds for the purpose of improving voice. For example, triamcinolone acetonide injection has been reported to improve voice in cases of chorditis, nodules, bamboo nodes, and polypoids [12]. Triamcinolone acetonide is a steroid preparation, and is mainly used to treat inflammation of the vocal folds in the otolaryngology voice field. On the other hand, Trafermin is bFGF preparation. bFGF, one of the body’s regulators, dissolves the vascular basement membrane [13] through the activation of plasminogen activator; this is a major angiogenic reaction that promotes the migration, proliferation, and lumen formation of vascular endothelial cells [14] as well as creation of capillaries in vivo [15, 16]. These effects are also known to cause the proliferation of fibroblasts [17], regeneration of muscle tissue [18], and lipogenesis [19]. Trafermin injections have been shown to improve voice in cases of vocal fold atrophy, scar, paralysis, and sulcus [7–9].

As a complication of intracordal injection under local anesthesia, edema of the vocal folds may cause airway emergencies. We have been performing more than 200 intracordal injections per year and have never experienced a case of tracheotomy or dyspnea after the injection. However, other complications may be encountered, such as cases of bleeding from the vocal folds when the drugs were injected into the vocal folds using an esophageal variceal needle. Continued bleeding from the vocal folds may cause blood to drip from the bronchial tubes into the lungs, causing swallowing and aspiration pneumonia. To prevent this, we dropped adrenaline onto the vocal folds directly after intracordal injection to stop bleeding. The adrenaline we used to stop the bleeding was Bosmin^®^ topical solution 0.1%, which is commonly used for the treatment of local bleeding, mucosal hyperemia, and swelling in otorhinolaryngology head and neck surgery. The dosage is not specified in the package insert, which states that the solution should be used as is or diluted five- to tenfold and applied directly or nasally, sprayed, or used as a tampon. The dose of adrenaline used for hemostasis in this study corresponds to 0.67 mg. On the other hand, an ampule of adrenaline kit (Adrenaline Injection^®^ 0.1% Syringe: Terumo Corporation Tokyo) administered intravenously to a patient in cardiopulmonary arrest contains 1 mg of adrenaline, which is at most 1.5 times the dose of adrenaline we use to stop bleeding for hemostasis after injection. However, we have had few cases of rapid vital changes after the injection, which led to the design of this study.

### Examination of endotracheal adrenaline administration dosage

The AHA [2] suggests that the endotracheal dose of adrenaline in patients with cardiopulmonary arrest should be 2–2.5 times the recommended intravenous dose; yet, the pharmacokinetics of endotracheal adrenaline is diverse, and the optimal dose is unknown. There are also reports that the pulmonary blood flow during cardiopulmonary arrest and cardiopulmonary resuscitation has been reported to be approximately 20% of the normal value, and the absorption of drugs through the alveoli is thought to be reduced [3]. Based on this paper, a simple calculation suggests that the dose of adrenaline administered in our injection is equivalent to 0.67 mg × 5 = 3.35 mg in the cardiopulmonary arrest. This is a higher dose than the 2–2.5 mg proposed by the AHA, yet no hemodynamic or vital changes occurred. Therefore, although the AHA recommends 2–2.5 mg of endotracheal adrenaline administration for cardiopulmonary arrest, we believe that a dose of 3.35 mg or more is necessary.

## Limitations

The limitations of this study are listed as follows. This was an observational study, which may have been susceptible to selection bias, information bias, and confounding factors. In addition, we did not report continuous heart rate and systolic blood pressure measurements because we used medical records to study heart rate and systolic blood pressure. Systolic blood pressure after adrenaline injection was measured approximately 1–3 min after the injection, but the blood pressure measurements may have been performed before or after the onset of adrenaline action. It might have been better to measure vital signs more often, with the sole purpose of ascertaining the patient's condition. In this study, triamcinolone acetonide and trafermin were injected, but the effects of these drugs on heart rate and systolic blood pressure have not been discussed. We used 4% Xylocaine as a local anesthetic, but its effects on heart rate and systolic blood pressure are also unknown. Adrenaline was administered directly to the vocal folds while the patient was vocalizing, but the actual proportion of the endotracheally administered 2 mL of adrenaline that was available to cause biological effects by actually reaching the alveoli may vary from case to case. Only 2 ml of adrenaline was administered, and other doses and concentrations could not be studied. Because the patients were not intubated in this study, the impact of positive pressure ventilation on drug dispersion and absorption after intubation was not discussed.

## Conclusion

In this study, no statistically significant changes were observed in the heart rate and systolic blood pressure in 79 patients who underwent intracordal injection under local anesthesia and received adrenaline endotracheally for hemostasis. We believe that the dose of adrenaline currently recommended by the AHA for endotracheal administration is too low to be used in patients with cardiopulmonary arrest. For medical ethical reasons, it is difficult to construct a study design in which adrenaline is administered endotracheally to human with non-cardiopulmonary arrest. Therefore, we believe that this study will provide useful information regarding the dosage of endotracheal adrenaline administration.

We, too, do not question the usefulness of intravenous and intraosseous adrenaline administration. In some cases of cardiopulmonary arrest, however, it is difficult to secure the peripheral venous pathway owing to a significant drop in blood pressure or inadequate peripheral circulation, and resuscitation opportunities may be missed. In addition, intraosseous routes of adrenaline administration requires a specialized needle and experience with the procedure. In some cases, such as limb disarticulation due to multiple traumatic injuries, intravenous and intraosseous administration may be difficult. In these cases, it is advisable to have many options for adrenaline administration. Therefore, if the optimal dose and efficacy of the endotracheal adrenaline administration are clarified, early adrenaline administration can be performed, which may improve the ROSC and survival discharge rates as well as intravenous and intraosseous administration. We will continue to investigate and analyze more cases, and hope that the findings of this study will be of value to clinicians in this regard.

## Data Availability

Yusuke Watanabe had full access to all the data in the study.
